# Serum periostin does not reflect type 2-driven inflammation in COPD

**DOI:** 10.1186/s12931-018-0818-8

**Published:** 2018-06-07

**Authors:** O. A. Carpaij, F. O. W. Muntinghe, M. B. Wagenaar, J. W. Habing, W. Timens, H. A. M. Kerstjens, M. C. Nawijn, L. I. Z. Kunz, P. S. Hiemstra, G. W. Tew, C. T. J. Holweg, C. A. Brandsma, M. van den Berge

**Affiliations:** 1 University of Groningen, University Medical Center Groningen, Groningen Research Institute for Asthma and COPD (GRIAC), Groningen, The Netherlands; 2 Department of Pulmonology, University of Groningen, University Medical Center Groningen, Hanzeplein 1, 9713 GZ Groningen, The Netherlands; 3 Department of Pathology and Medical Biology, University of Groningen, University Medical Center Groningen, Groningen, The Netherlands; 4 Department of Pulmonology, Leiden University Medical Center, Leiden, The Netherlands; 5 Genentech, Inc., OMNI Biomarker Development, South San Francisco, California, USA

**Keywords:** Serum periostin, COPD, Biomarker, Type 2-inflammation, Inhaled corticosteroid response

## Abstract

Although Th2 driven inflammation is present in COPD, it is not clearly elucidated which COPD patients are affected. Since periostin is associated with Th2 driven inflammation and inhaled corticosteroid (ICS)-response in asthma, it could function as a biomarker in COPD. The aim of this study was to analyze if serum periostin is elevated in COPD compared to healthy controls, if it is affected by smoking status, if it is linked to inflammatory cell counts in blood, sputum and endobronchial biopsies, and if periostin can predict ICS-response in COPD patients.

Serum periostin levels were measured using Elecsys Periostin immunoassay. Correlations between periostin and inflammatory cell count in blood, sputum and endobronchial biopsies were analyzed. Additionally, the correlation between serum periostin levels and treatment responsiveness after 6 and 30 months was assessed using i.e. ΔFEV1% predicted, ΔCCQ score and ΔRV/TLC ratio. Forty-five COPD smokers, 25 COPD past-smokers, 22 healthy smokers and 23 healthy never-smokers were included. Linear regression analysis of serum periostin showed positive correlations age (B = 0.02, 95%CI 0.01–0.03) and FEV1% predicted (B = 0.01, 95%CI 0.01–0.02) in healthy smokers, but not in COPD patients In conclusion, COPD -smokers and -past-smokers have significantly higher periostin levels compared to healthy smokers, yet periostin is not suitable as a biomarker for Th2-driven inflammation or ICS-responsiveness in COPD.

**Electronic supplementary material:**

The online version of this article (10.1186/s12931-018-0818-8) contains supplementary material, which is available to authorized users.

## To the editor

Recent research suggests that type 2-driven eosinophilic inflammation is present in a subset of COPD patients [1]. This is important as it may predict responsiveness to anti-inflammatory treatment with inhaled corticosteroids (ICS) and possibly also targeted therapies like interleukin-5 monoclonal antibodies [2].

Periostin is an extracellular matrix protein that has been proposed as biomarker for type 2-driven inflammation [3]*.* While the majority of studies so far investigated the clinical implication of circulating periostin levels in asthma, data regarding COPD is scarce [3–5].

The aim of this study was to investigate whether serum periostin levels are different in COPD patients compared to healthy controls and whether they are affected by smoking. In addition, we assessed to what extent serum periostin levels reflect inflammatory cell counts in blood, sputum and endobronchial biopsies in COPD and whether serum periostin levels predict airway wall remodeling and ICS responsiveness following treatment of 6 or 30 months.

We included COPD patients who participated in the Groningen and Leiden Universities study of Corticosteroids in Obstructive Lung Disease (GLUCOLD) as described previously [6, 7]. Patients were 45–75 years, Caucasian, had an FEV_1_/FVC ratio < 70%, ≥10 pack years, and no history of asthma. Subjects were randomly assigned to receive long-term ICS with or without an added long-acting beta2-agonist (LABA) or placebo-treatment.

Healthy subjects were 40–75 years, Caucasian, had an FEV_1_/FVC ratio ≥ 70% and PC_20_ methacholine > 19.6 mg/mL [8], and were divided into smokers (i.e. smoking ≥10 cigarettes/day and ≥ 10 pack years) and never-smokers.

From the previous studies [6, 8], COPD patients and healthy controls underwent the following tests: pulmonary function tests, peripheral blood tests, sputum induction, a bronchoscopy and filled in questionnaires. From the present study, serum periostin levels were measured using the clinical trial version of the Elecsys® Periostin immunoassay (Roche Diagnostics, Penzberg Germany) [9].

The local ethics committee approved both study protocols and all subjects gave written informed consent.

First, demographic and clinical variables in COPD patients and healthy controls were compared using independent sample t-tests for normally distributed data, Mann-Whitney U tests for non-normally distributed data and chi-square tests for categorical variables. To assess possible confounders of log2 transformed periostin values, a univariate analysis was performed. Next, a linear regression model was used to assess the association between serum periostin levels and inflammatory cell counts in blood, sputum and endobronchial biopsies at baseline, with correction for significant confounders. A linear regression was used to analyze serum periostin levels in association with ICS treatment responsiveness (i.e. change in FEV_1_, Clinical COPD Questionnaire (CCQ)-total score and RV/TLC after 6 and 30 months treatment) and airway wall remodelling in COPD patients. Airway wall remodeling in endobronchial biopsies was measured by dividing immunohistochemical stained area for elastic fibers, versican, decorin, collagen I and III by the total selected lamina propria area as described previously [7].

Of the 114 COPD patients enrolled in GLUCOLD, 70 subjects had available measured serum periostin level at baseline. COPD smokers (*n* = 45/70) were 60.3 (SD ± 7.9) years, smoked 46.7 (SD ± 19.8) packyears, had a % FEV_1_ predicted of 63.8% (SD ± 7.8%) and a serum periostin level of 51.8 ng/ml [IQR 48.4–59.8 ng/ml]. COPD former-smokers (*n* = 25/70) had a mean age of 64.7 (SD ± 7.3), smoked 45.6 (SD ± 27.7) pack years, % FEV_1_ predicted of 60.9% (SD ± 10.5%) and a serum periostin level of 54.8 ng/ml [IQR 47.8–62.2 ng/ml]. The healthy smokers (*n* = 22) were 52.1 (SD ± 7.5) years, smoked 29.0 (SD ± 11.6) pack years, had a mean % FEV_1_ predicted of 104.0% (SD ± 11.2%) and a serum periostin of 44.6 ng/ml [IQR 39.8–51.2 ng/ml]. The healthy never-smokers (*n* = 23) had a mean age of 58.4 (SD ± 9.1), mean FEV_1_% predicted of 108.6% (SD ± 13.9%) and serum periostin of 49.7 ng/ml [IQR 41.8–54.7 ng/ml] (see Additional file 1).

Serum periostin was significantly higher in COPD smokers (*P* = 0.009) as well as COPD former-smokers (*P* = 0.001) compared to the healthy smoker-group. Serum periostin was similar between COPD smokers and COPD former-smokers. In agreement with our findings, Golpe et al. also found higher significantly periostin levels in both tobacco smoke- as well as biomass cooking-induced COPD compared to healthy controls [10]. However, the latter study only investigated never-smoking controls and did not include matched current- and former-smoking controls. In addition, they used another method to analyse serum periostin and found undetectable levels in the never-smoking controls. Two other studies did not find a difference in serum periostin levels between COPD and predominantly never-smoker controls [4, 5].

In our study, healthy never-smokers tended to have higher periostin levels compared to healthy smokers (*P* = 0.08), which was also seen in other studies [11, 12]. Caswell-Smith et al. saw a significantly higher periostin level in 312 never-smokers compared to 22 healthy smokers [12]. Taking together, there is evidence that current smoking is associated with lower serum periostin levels in healthy controls. It is interesting to note that studies from our laboratories using cultured human bronchial epithelial cells did not provide evidence for induction of epithelial periostin expression by cigarette smoke exposure, an even showed that type 2 cytokine (IL-13) induced periostin expression was suppressed by cigarette smoke [13].

Next, we assessed the correlations between serum periostin levels and clinical and inflammatory characteristics in COPD patients and healthy smokers and never-smokers. Results of the univariate and linear regression analyses are presented in Table 1. In COPD patients, no correlation was found between periostin and age, lung function and inflammatory cell counts in blood, sputum and biopsies. In the healthy smoker-group, periostin levels were significantly positively associated with age (B = 0.02, 95%CI 0.01–0.03) and post-bronchodilator FEV_1_% predicted (B = 0.01, 95%CI 0.01–0.02). After adjusting the data for the last mentioned possible confounders, no further correlations were found in the healthy smokers. In the healthy never-smoker group, periostin was associated with higher percentages of sputum lymphocytes (B = 0.3, 95%CI 0.1–0.5). Our finding is that serum periostin levels do not reflect type 2-driven inflammation in COPD, is in agreement with the findings of Konstantelou et al. who measured serum periostin in 155 COPD patients admitted for a COPD exacerbation and found no correlations with severity of airflow obstruction or eosinophilic inflammation measured in blood [14].

**Table 1:** Regression analysis of baseline log2 transformed periostin with baseline characteristics in COPD-patients, healthy smokers and never-smokers

	ICS naive COPD	Healthy smoker	Healthy never-smoker
	(*n* = 70)	(*n* = 22)	(*n* = 23)
Univariate regression	**B**_**exp**_ **(95% CI)**	**B**_**exp**_ **(95% CI)**	**B**_**exp**_ **(95% CI)**
Sex, male (%)	4.7 (0.6–36.9)	0.2 (0.004–11.4)	1.9 (0.1–29.7)
Smokers (%)	0.4 (0.1–2.0)	NA	NA
Linear regression	**B (95% CI)**	**B (95% CI)**	**B (95% CI)**
Packyears (years)	7.0 × 10^− 5^ (− 0.004–0.004)	NA	NA
Age (years)	0.01 (− 0.003–0.2)	0.02 (0.01–0.03) *	0.01 (− 0.01–0.03)
BMI, (kg/m^2^)	0.2 (− 0.1–0.1)	−0.1 (− 0.3–0.1)	0.04 (− 0.1–0.2)
% predicted FEV_1_ post-bronchodilator	−0.03 (− 0.01–0.01)	0.01 (0.04–0.02) *	0.01 (− 0.01–0.02)
FEV_1_/IVC ratio (%)	0.01 (− 0.004–0.01)	0.0 (− 0.02–0.02)	0.013 (− 0.02–0.04)
RV/TLC ratio (%)	0.003 (− 0.01–0.01)	0.02 (− 0.02–0.1)	0.02 (− 0.01–0.04)
Fractional exhaled Nitric Oxide (ppb)	0.001 (− 0.01–0.01)	NA	NA
Total IgE (IU/L)	4.2 × 10^− 5^ (0.0–0.0)	NA	NA
PC_20_ methacholin threshold (mg/ml) ^#^	0.1 (− 0.03–0.1)	NA	NA
Blood eosinophils (%) ^#^	0.1 (− 0.03–0.1)	− 0.07 (− 0.2–0.1)	0.03 (− 0.2–0.2)
Blood basophils (%) ^#^	0.04 (− 0.04–0.1)	−0.1 (− 0.2–0.1)	0.1 (− 0.1–0.2)
Blood neutrophils (%)	−0.001 (− 0.01–0.01)	− 0.004 (− 0.02–0.01)	−0.02 (− 0.04–0.01)
Blood monocytes (%)	0.03 (− 0.01–0.1)	0.01 (− 0.1–0.1)	0.02 (− 0.08–0.1)
Blood lymphocytes (%)	−0.003 (− 0.01–0.01)	0.01 (− 0.01–0.02)	0.02 (− 0.01–0.04)
Sputum eosinophils (%)	− 0.01 (− 0.1–0.04)	−0.01 (− 0.2–0.1)	0.1 (− 0.1–0.2)
Sputum neutrophils (%)	−7.7 × 10^− 6^ (− 0.01–0.01)	0.002 (− 0.006–0.01)	−0.004 (− 0.01–0.01)
Sputum macrophages (%)	−0.001 (− 0.01–0.01)	−0.002 (− 0.01–0.01)	0.001 (− 0.01–0.01)
Sputum lymphocytes (%)	0.1 (− 0.01–0.1)	0.108 (− 0.2–0.4)	0.3 (0.1–0.5) *
Biopsy eosinophils (count/0.1mm^2^)	−0.001 (− 0.003–0.002)	−0.03 (− 0.1–0.06)	0.01 (− 0.03–0.04)
Biopsy neutrophils (count/0.1mm^2^) ^#^	0.02 (− 0.03–0.08)	−0.002 (− 0.07–0.07)	−0.08 (− 0.2–0.04)
Biopsy macrophages (count/0.1mm^2^) ^#^	0.045 (− 0.03–0.1)	−0.03 (− 0.1–0.1)	0.03 (− 0.1–0.1)
Biopsy lymphocytes (count/0.1mm^2^)	0.000 (− 0.001–0.001)	−0.01 (− 0.01–0.002)	−0.02 (− 0.1–0.1)
Biopsy elastic fibers area (%)	0.002 (− 0.01–0.01)	NA	NA
Biopsy elastic fibers density (gray value)	0.002 (− 0.01–0.02)	NA	NA
Biopsy versican area (%)	−0.001 (− 0.01–0.01)	NA	NA
Biopsy versican density (gray value)	−0.01 (− 0.03–0.01)	NA	NA
Biopsy decorin area (%)	− 8.15 × 10^− 6^ (< 0.001 – < 0.001)	NA	NA
Biopsy decorin density (gray value)	−0.003 (− 0.1–0.045)	NA	NA
Biopsy collagen I area (%)	−0.003 (− 0.01–0.01)	NA	NA
Biopsy collagen I density (gray value)	0.003 (− 0.02–0.03)	NA	NA
Biopsy collagen III area (%)	−0.004 (− 0.01–0.03)	NA	NA
Biopsy collagen III density (gray value)	−0.01 (− 0.02–0.01)	NA	NA
Biopsy mean number of ki-67 pos. Cells (count/0.1mm^2^)	0.001 (− 0.001–0.003)	NA	NA
Biopsy PAS pos. Area epithelium (%)	0.000 (− 0.01–0.01)	NA	NA
Biopsy EGFR pos. Epithelium area (%)	0.002 (− 0.01–0.01)	NA	NA
Biopsy EGFR pos. Epithelium density (gray value)	5.8 × 10^− 6^ (< 0.001 – < 0.001)	NA	NA

#: log2 transformed variable, *: statistically significant P < 0.05, BMI: Body Mass Index. NA: not available, area (%): the percentage stained area for a specific extracellular matrix component was calculated dividing the stained area by the total selected area, density (gray value): staining intensity was analyzed by densitometry (weighted mean per biopsy) and presented as gray value (black: gray value: 0, white: gray value: 255)

To our analysis, baseline periostin levels did not predict ICS responsiveness in COPD; there was no correlation with improvement in lung function, decrease in hyperinflation or CCQ-total score after either 6 or 30 months of ICS-treatment. Studies investigating periostin as biomarker for ICS treatment in COPD patients are limited. In this context, the findings of Park et al. are of interest [15]. They studied 130 COPD patients before and after three months of ICS/LABA treatment and found that a combination of high plasma periostin levels (> 23 ng/mL) and high blood eosinophil counts (> 260/μL) could predict a better improvement in FEV_1_. However, it is important to note that patients with this combination of high periostin and blood eosinophils already had a higher bronchodilator response at baseline and therefore the better improvement might have been due to the LABA component alone.

Finally, no correlation was detected between baseline periostin and change in extracellular matrix (lamina propria components stained area or density) after 30 months on ICS or placebo treated COPD patients (see Additional file 1).

## Conclusion

In conclusion, we show that smoking and former-smoking COPD patients have significantly higher serum periostin values compared to healthy smoking controls, yet periostin levels do not reflect type 2-driven inflammation, airway remodeling, or ICS treatment responsiveness and is thus not a good biomarker in this population.

## Additional file

Additional file 1: Supplementary Tables. (DOCX 95 kb)

## Abbreviations

CCQ: Clinical COPD Questionnaire
GLUCOLD: Groningen and Leiden Universities study of Corticosteroids in Obstructive Lung Disease
ICS: Inhaled corticosteroids
LABA: Long-acting beta2-agonist
