# Supplementary material for: Serum periostin does not reflect type 2-driven inflammation in COPD
Source: Respir Res. 2018 Jun 7;19:112. doi: 10.1186/s12931-018-0818-8 (PMC5992772; doi:10.1186/s12931-018-0818-8)
Supplement: Supplementary file 1 — Supplementary Tables. (DOCX 95 kb) [file 12931_2018_818_MOESM1_ESM.docx]

| **Supplementary table 1: baseline characteristics of COPD smokers, COPD former-smokers, healthy smokers and healthy never-smokers** | | | | | |
| --- | --- | --- | --- | --- | --- |
|  | | **COPD smokers**  **(n = 45)** | **COPD former-smokers (n = 25)** | **Healthy smokers**  **(n = 22)** | **Healthy never-smokers (n = 23)** |
|  | Sex, male (%) | 37 (82.2%) | 24 (96.0%) | 13 (59.1%) | 16 (69.6%) |
|  | Age (years) | 60.3 ± 7.9 ^a b^ | 64.7 ± 7.3 ^a^ | 52.1 ± 7.5 ^b c^ | 58.4 ± 9.1 ^c^ |
|  | BMI (kg/m^2^) | 25.2 ± 4.2 | 26.4 ± 3.5 | 24.7 ± 3.2 | 25.6 ± 4.4 |
|  | Packyears (years) | 46.7 ± 19.8 ^b^ | 45.6 ± 27.7 | 29.0 ± 11.6 ^b c^ | NA ^c^ |
| PFT | PC_20_ methacholin threshold (mg/ml) ^#^ | 0.9 [0.2 – 2.3] | 0.3 [0.1 – 1.4] | NA | NA |
|  | Predicted post-bronchodilator FEV_1_ (%) | 63.8 ± 7.8 ^b^ | 60.9 ± 10.5 | 104.0 ± 11.2 ^b^ | 108.7 ± 13.9 |
|  | FEV_1_/IVC ratio (%) | 45.2 [40.7 – 53.5] ^b^ | 45.1 [37.8 – 52.2] | 73.5 [69.8 – 76.7] ^b^ | 73.4 [70.9 – 76.1] |
|  | RV/TLC ratio (%) | 49.3 ± 7.9 ^b^ | 45.6 ± 7.7 | 30.1 ± 2.7 ^b^ | 31.2 ± 5.4 |
|  | Fe_­­_NO (ppb) | 4.8 [3.9 – 8.4] ^a^ | 14.9 [9.3 – 19.6] ^a^ | NA | NA |
| Blood | Total IgE (IU/L) | 131.1 ± 265.1 | 164.7 ± 272.8 | NA | NA |
|  | Periostin (ng/ml) ^#^ | 51.8 [48.4 – 59.8] ^b^ | 54.8 [47.8 – 62.2] | 44.6 [39.8 – 51.2] ^b^ | 49.7 [41.8 – 54.7] |
|  | ≥75^th^ percentile Periostin (≥55.4 ng/ml) (%) | 17 (37.8%) ^b^ | 12 (48.0%) | 1 (4.5%) ^b^ | 5 (21.7%) |
|  | Eosinophils (%) ^#^ | 2.2 [1.3 – 3.4] | 2.8 [1.4 – 3.9] | 2.2 [1.7 – 3.1] | 2.2 [1.5 – 3.6] |
|  | Basophils (%) ^#^ | 0.5 [0.3 – 0.7] | 0.5 [0.3 – 0.8] | 0.4 [0.2 – 0.6] ^c^ | 0.6 [0.4 – 1.0] ^c^ |
|  | Neutrophils (%) | 58.8 ± 7.2 | 57.5 ± 12.7 | 57.7 ± 8.8 | 54.9 ± 6.4 |
|  | Monocytes (%) | 8.8 ± 2.4 | 9.1 ± 2.6 | 8.2 ± 1.9 | 7.4 ± 1.6 |
|  | Lymphocytes (%) | 29.2 ± 7.0 | 29.9 ± 11.3 | 31.2 ± 7.7 | 34.3 ± 5.6 |
| Sputum | Eosinophils (%) | 1.0 [0.3 – 2.2] | 1.3 [0.3 – 2.5] | 0.4 [0.2 – 0.9] ^c^ | 0.0 [0.0 – 0.3] ^c^ |
|  | Basophils (%) | 0.0 [0.0 - 0.0] | 0.0 [0.0 – 0.0] | 0.0 [0.0 - 0.0] | 0.0 [0.0 – 0.0] |
|  | Neutrophils (%) | 66.2 [49.6 – 73.1] ^a b^ | 73.2 [64.1 – 75.4] ^a^ | 50.0 [41.7 – 69.7] ^b^ | 45.7 [34.1 – 60.9] |
|  | Macrophages (%) | 28.2 [21.3 – 39.0] ^a b^ | 22.0 [18.1 – 28.8] ^a^ | 44.3 [26.9 – 55.5] ^b^ | 47.0 [34.6 – 62.0] |
|  | Lymphocytes (%) | 1.7 [1.2 – 2.2] ^a b^ | 2.3 [1.9 – 4.0] ^a^ | 0.4 [0.0 – 0.8] ^b^ | 0.7 [0.3 – 0.9] |
| Biopsy | Eosinophils (count / 0.1mm^2^) | 1.0 [0.5 – 4.0] ^b^ | 2.0 [0.5 – 5.5] | 0.8 [0.0 – 1.5] ^b^ | 0.8 [0.0 – 2.3] |
|  | Neutrophils (count / 0.1mm^2^) ^#^ | 4.0 [1.5 – 7.5] | 5.0 [2.0 – 8.8] | 1.7 [0.7 – 5.0] ^c^ | 7.1 [3.5 – 11.2] ^c^ |
|  | Macrophages (count / 0.1mm^2^) ^#^ | 8.5 [4.5 – 12.0] | 10.5 [5.3 – 13.3] | 4.9 [1.5 – 14.3] | 7.1 [2.8 – 12.3] |
|  | Lymphocytes (count / 0.1mm^2^) | 109.0 [61.8 – 167.8] ^b^ | 169.5 [79.8 – 220.8] | 21.1 [12.7 – 37.5] ^b^ | 30.9 [17.0 – 41.7] |
| *Data is presented as mean* ± standard deviation, *median [IQR] or dichotomous (%), #: log2 transformed and presented in geometric mean [original IQR],*  *a: statistical significance (P <.05) between smoking COPD and ex-smoking COPD group, b: statistical significance (P <.05) between smoking COPD and healthy smoker group, c: statistical significance (P <.05) between healthy smoker and healthy never-smoker group, ICS: inhaled corticosteroids, BMI: Body Mass Index, FEV_1_ pred. %: FEV_1_ as percentage of the predicted value, NA: not available.* | | | | | |

**Supplementary figure 2**


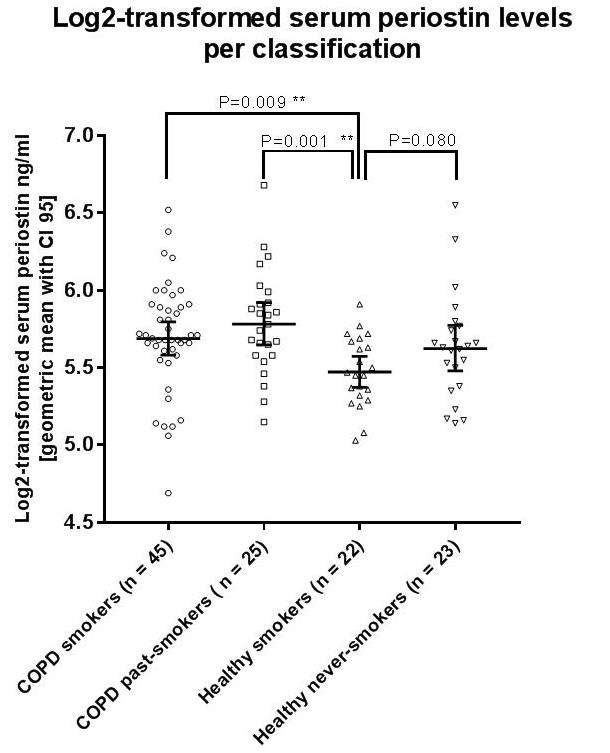


| **Supplementary table 2A: linear regression analysis of serum periostin level and clinical parameters in COPD patients with and without ICS-treatment** | | |
| --- | --- | --- |
| **ICS treatment group** | | |
|  | **6 vs. 0 months (n = 77)** | **30 vs. 0 months (n = 51)** |
|  | B (95% CI) | B (95% CI) |
| ΔFEV_1_ | 0.1 (0.03 – 0.3) | 0.2 (-0.1 – 0.7) |
| ΔRV/TLC | 0.01 (-0.1 – 0.2) | 0.6 (-0.01 – 1.3) |
| ΔCCQ | 0.01 (-0.01 – 0.03) | 0.01 (-0.01 – 0.03) |
| **Placebo group** | | |
|  | **6 vs. 0 months (n = 24)** | **30 vs. 0 months (n = 24)** |
|  | B (95% CI) | B (95% CI) |
| ΔFEV_1_ | -0.01 (-0.2 – 0.1) | 0.1 (-0.08 – 0.2) |
| ΔRV/TLC | 0.01 (-0.1 – 0.2) | -0.2 (-0.5 – 0.2) |
| ΔCCQ | 0.002 (-0.01 – 0.1) | 0.01 (-0.01 – 0.02) |
| *ICS: inhaled corticosteroids, ΔFEV_1_: change in FEV_1_ after 6 and 30 months,   univariate analysi g ntealthy. atients (GOLD I and II), and healthy past-smoker controls. ΔRV/TLC: change in RV/TLC ratio (residual volume divided by total lung capacity) after 6 and 30 months, ΔCCQ: change in CCQ questionnaire score defined after 6 and 30 months.* | | |

| **Supplementary table 2B: linear regression analysis of log 2 transformed serum periostin level and clinical parameters in COPD patients with and without ICS-treatment** | | |
| --- | --- | --- |
| **ICS treatment group** | | |
|  | **6 vs. 0 months (n = 77)** | **30 vs. 0 months (n = 51)** |
|  | B (95% CI) | B (95% CI) |
| ΔFEV_1_ | 0.01 (-0.01 – 0.03) | 0.004 (-0.01 – 0.02) |
| ΔRV/TLC | 0.001 (-0.01 – 0.01) | 0.002 (-0.01 – 0.01) |
| ΔCCQ | 0.1 (-0.01 – 0.3) | 0.08 (-0.1 – 0.3) |
| **Placebo group** | | |
|  | **6 vs. 0 months (n = 24)** | **30 vs. 0 months (n = 24)** |
|  | B (95% CI) | B (95% CI) |
| ΔFEV_1_ | -3.6 x 10^-5^ (-0.1 – 0.1) | -0.01 (-0.1 – 0.1) |
| ΔRV/TLC | -0.002 (-0.02 – 0.02) | -0.02 (-0.04 – 0.001) |
| ΔCCQ | 0.1 (-0.6 – 0.8) | 0.2 (-0.5 – 0.8) |
| *ICS: inhaled corticosteroids, ΔFEV_1_: change in FEV_1_ after 6 and 30 months, ΔRV/TLC: change in RV/TLC ratio (residual volume divided by total lung capacity) after 6 and 30 months, ΔCCQ: change in CCQ questionnaire score defined after 6 and 30 months.* | | |

| **Supplementary table 3A: linear regression analysis of serum periostin and 30 months change in lamina propria component area/density in COPD patients with and without ICS-treatment** | | |
| --- | --- | --- |
|  | **Placebo group (n = 24)** | **ICS treatment group (n = 51)** |
| Δ Elastic fibers area (%) | -0.04 (-1.4 – 1.3) | 0.5 (-1.0 – 2.0) |
| Δ Elastic fibers density (gray value) | -0.07 (-0.8 – 0.6) | 0.4 (-0.06 – 1.0) |
| Δ Versican area (%) | 0.8 (-0.1 – 1.8) | 0.1 (-1.1 – 1.3) |
| Δ Versican density (gray value) | 0.4 (-0.01 – 0.8) | 0.1 (-0.3 – 0.4) |
| Δ Decorin area (%) | 0.04 (-0.3 – 0.4) | -0.03 (-1.0 – 0.9) |
| Δ Decorin density (gray value) | 0.1 (-0.4 – 0.6) | 0.01 (-0.5 – 0.5) |
| Δ Collagen I area (%) | 0.2 (-0.9 – 1.3) | 0.3 (-1.6 – 2.1) |
| Δ Collagen I density (gray value) | 0.3 (-0.4 – 0.4) | -0.04 (-0.6 – 0.5) |
| Δ Collagen III area (%) | 0.1 (-2.4 – 2.6) | -0.2 (-2.2 – 1.7) |
| Δ Collagen III density (gray value) | -0.3 (-2.2 – 1.6) | -0.3 (-1.1 – 0.5) |
| *Δ: Change after 30 months, area (%): the percentage stained area for a specific extracellular matrix component was calculated dividing the stained are by the total selected area, density (gray value): staining intensity was analyzed by densitometry (weighted mean per biopsy) and presented as gray value (black: gray value: 0, white: gray value: 255). No statistical correlation was found (P<0.05).* | | |

| **Supplementary table 3B: linear regression analysis of log 2 transformed serum periostin and 30 months change in lamina propria component area/density in COPD patients with and without ICS-treatment** | | |
| --- | --- | --- |
|  | **Placebo group (n = 24)** | **ICS treatment group (n = 51)** |
| Δ Elastic fibers area (%) | -0.001 (-0.02 – 0.02) | 0.004 (-0.01 – 0.02) |
| Δ Elastic fibers density (gray value) | -0.005 (-0.05 – 0.04) | 0.02 (-0.01 – 0.046) |
| Δ Versican area (%) | 0.02 (-0.01 – 0.04) | 0.0 (-0.02 – 0.02) |
| Δ Versican density (gray value) | 0.04 (-0.004 – 0.1) | -0.01 (-0.047 – 0.1) |
| Δ Decorin area (%) | 0.01 (-0.1 – 0.1) | 0.0 (-0.02 – 0.02) |
| Δ Decorin density (gray value) | 0.01 (-0.1 – 0.1) | 0.01 (-0.1 – 0.2) |
| Δ Collagen I area (%) | 0.01 (-0.03 – 0.04) | 0.002 (-0.01 – 0.01) |
| Δ Collagen I density (gray value) | 0.01 (-0.1 – 0.1) | -0.001 (-0.04 – 0.04) |
| Δ Collagen III area (%) | 0.001 (-0.01 – 0.02) | -0.002 (-0.01 – 0.01) |
| Δ Collagen III density (gray value) | -0.003 (-0.02 – 0.02) | -0.01 (-0.03 – 0.01) |
| *Δ: Change after 30 months, area (%): the percentage stained area for a specific extracellular matrix component was calculated dividing the stained are by the total selected area, density (gray value): staining intensity was analyzed by densitometry (weighted mean per biopsy) and presented as gray value (black: gray value: 0, white: gray value: 255). No statistical correlation was found (P<0.05).* | | |
